# Bioluminescence in the Edible Mushroom *Hypsizygus marmoreus* by Transformation with a Fungal Luciferase Gene

**DOI:** 10.3390/jof12060417

**Published:** 2026-06-09

**Authors:** Xinyu Zhou, Yan Li, Yingying Wu, Ruisheng Chen, Lihua Tang, Chenli Zhou, Jianing Wan, Dapeng Bao, Ruiheng Yang, Junjun Shang

**Affiliations:** 1 Key Laboratory of Edible Fungi Resources and Utilization (South), Ministry of Agriculture, National Engineering Research Center of Edible Fungi, National R&D Center for Edible Fungi Processing, Institute of Edible Fungi, Shanghai Academy of Agricultural Sciences, Shanghai 201403, China; 2 Institute of Plant Protection, Jilin Agricultural University, Changchun 130118, China; 3 Institute of Mycology, Jilin Agricultural University, Changchun 130118, China; 4 College of Food Science, Shanghai Ocean University, Shanghai 201306, China

**Keywords:** fungal luciferase gene, Bioluminescence, *Hypsizygus marmoreus*, *Agrobacterium*-mediated transformation

## Abstract

Following the elucidation of the fungal bioluminescence pathway (FBP), it was quickly adopted as a reporter system in plants; however, no such application has been documented in fungi to date. In this study, we established for the first time a luminescent reporter in the commercially important mushroom *Hypsizygus marmoreus* by expressing the luciferase gene from the luminous fungus *Neonothopanus nambi.* Using an established *Agrobacterium*-mediated transformation method, we separately introduced the wild-type luciferase gene *nnLuz* and the previously reported optimized variant *nnLuz-v4* that can enhance bioluminescence expression into *H. marmoreus* arthroconidia. Both genes were stably integrated into the genome and expressed under the control of the *H. marmoreus* Glycerol 3-phosphate dehydrogenase (GPD) gene promoter. Upon addition of exogenous luciferin, transformants carrying the wild-type *nnLuz* produced clear, readily detectable bioluminescence signals, whereas no luminescence was observed in untransformed controls. Unexpectedly, the wild-type luciferase consistently exhibited substantially higher luminescence intensity than the optimized *nnLuz-v4* variant. This finding suggests that codon optimization may be unnecessary or even detrimental when the donor and host are phylogenetically close basidiomycetes. The successful deployment of the fungal luciferase gene in *H. marmoreus* provides a sensitive and non-invasive genetic tool that does not require external excitation. This system opens new avenues for promoter characterization, real-time gene expression monitoring during mushroom development, and molecular breeding efforts aimed at improving agronomically important traits.

## 1. Introduction

The natural phenomenon of mushroom mycelium or cap emitting visible light at night has been recognized for centuries, yet the underlying biochemical mechanism remained elusive for a long time. A landmark discovery was made in 1959 when luciferase and its luciferin substrate were extracted from luminescent fungi [1]. However, it took nearly six decades to elucidate the complete bioluminescence pathway. In 2018, Kotlobay et al. revealed the molecular circuit of fungal bioluminescence, identifying the key enzymes and the cyclic mechanism of fungal luciferin regeneration [2].

Shortly after the disclosure of the fungal bioluminescence pathway (FBP), the first successful engineering of autonomous luminescent plants using the fungal luciferase system was reported in 2020 [3]. This breakthrough was subsequently adopted by several laboratories for plant synthetic biology applications [4,5]. Nevertheless, inconsistent results emerged, with some studies reporting no or extremely weak luminescence in certain plant species [6]. To address these limitations, a major optimization was achieved in 2024: the luciferase-encoding gene was subjected to codon optimization for plants, yeast, and human expression systems, combined with site-directed mutagenesis in bacteria. The resulting enhanced fungal luciferase system increased luminescence intensity by one to two orders of magnitude [7].

In parallel, edible mushrooms are cultivated on an industrial scale, and genetic improvement is of paramount importance for yield and quality. Mushroom genetics research serves as the foundation for such breeding efforts. Fluorescent markers, particularly GFP, have been widely used as genetic tools in edible mushroom research [8,9]. However, GFP requires blue light excitation and an expensive fluorescence microscope, limiting its convenience and throughput. Interestingly, although the FBP has been successfully repurposed for studying gene expression and promoter activity in plants [10], and despite the close phylogenetic relationship between most edible mushrooms (order Agaricales) and luminescent fungi (also within Agaricales), there has been no report of using the FBP as a reporter system in edible mushroom genetics.

*Hypsizygus marmoreus* is a commercially important edible mushroom cultivated on a large scale in China. Our laboratory has been dedicated to improving *H. marmoreus* strains through molecular genetic approaches [11,12]. A convenient, highly sensitive, and non-invasive tracer marker is essential for studying gene function and genetic regulation in this species. In the present study, we leveraged our previously established *Agrobacterium*-mediated transformation method using arthroconidia as recipient material [13]. We separately transformed the wild-type luciferase gene *nnLuz* from the luminescent mushroom *Neonothopanus nambi* and the final optimized version *nnLuz-v4* [7] into *H. marmoreus*. Upon addition of the luciferin substrate, luminescence signals were detected with high sensitivity. This new tool enables robust investigation of gene expression patterns and promoter dynamics, thereby empowering molecular breeding of *H. marmoreus*.

## 2. Materials and Methods

### 2.1. Strains and Culture Conditions

The wild-type *H. marmoreus* dikaryotic strain HZ40 used in this study was maintained in our laboratory and cultivated on potato dextrose agar (PDA) at 25 °C. Arthroconidia were harvested from 14-day-old cultures grown on PDA plates according to the method described by Bao et al., which utilises arthroconidia as recipient cells for genetic transformation [13]. *Escherichia coli* strain DH5α was used for plasmid construction, and *Agrobacterium tumefaciens* strain EHA105 was used for fungal transformation.

### 2.2. Vector Construction

The fungal luciferase gene sequence was sourced from the published FBP of *Neonothopanus nambi* [2]. Two versions of the gene were commercially synthesised: the wild-type sequence (referred to as *nnLuz*) and the optimized version (referred to as *nnLuz-v4*) [7]. Both sequences were cloned into the binary vector pCAMBIA1300, in which expression was driven by the *H. marmoreus* Glycerol 3-phosphate dehydrogenase (GPD) gene promoter [14]. The carboxin resistance gene (*Cbx^R^*) from *Pleurotus eryngii* served as the selectable marker under the control of its native promoter [15].

### 2.3. Agrobacterium-Mediated Transformation of Arthroconidia

*Agrobacterium*-mediated transformation (ATMT) of *H. marmoreus* arthroconidia was performed following the optimized protocol reported for this species [13]. Briefly, *A. tumefaciens* EHA105 cells harbouring the respective binary vectors were cultured in induction medium containing 200 μM acetosyringone to an OD_600_ of 0.4–0.6. Freshly harvested *H. marmoreus* arthroconidia were co-cultivated with induced *Agrobacterium* cells on nitrocellulose membranes overlaid on co-cultivation agar plates at 28 °C for two days. Following co-cultivation, the membranes were transferred to selection medium containing 2 μg/mL carboxin and 300 μg/mL cefotaxime to suppress residual *Agrobacterium* growth. Carboxin-resistant colonies that emerged after 10–14 days were subcultured onto fresh selective medium for three successive rounds to ensure mitotic stability.

### 2.4. Molecular Confirmation of Transformants

Genomic DNA was extracted from carboxin-resistant colonies using the cetyltrimethyl ammonium bromide (CTAB) method. Integration of the transgene was confirmed by PCR amplification with primers specific to the luciferase gene sequences. The PCR primers for *nnLuz* were nnluz-F3 (5′-ACCAGACTTTCCTAGAAGTG-3′) and nnluz-R3 (5′-AGATGAAGTGTGCAATCATG-3′), with an expected amplicon size of 457 bp. The PCR primers for *nnLuz-v4* were nnluz-F5 (5′-GGAGAGACTACCAGACCTTC-3′) and nnluz-R5 (5′-GTCCTCCTGACAGTATCATG-3′), with an expected amplicon size of 415 bp. PCR products were resolved by agarose gel electrophoresis and visualized under UV illumination after ethidium bromide staining.

### 2.5. Luminescence Imaging

For luminescence detection, mycelial plugs were taken from PDA plates on which mycelium of wild-type and transformants had grown, placed in an empty Petri dish, and 1 mmol/L luciferin was added onto the mycelial surface. Luminescence was captured using the Tanon 5200 automatic chemiluminescence image analysis system equipped with a Sony ICX694 CCD camera (Tanon, Shanghai, China). All imaging experiments were performed at least three times independently, with representative images presented.

### 2.6. Image Processing and Statistical Analysis

The online image processing software ImageJ 1.53m (https://ij.imjoy.io/) was used to quantify the luminescence intensity of different strains in images taken after luciferin addition. The GraphPad Prism 9 software was used for statistical analysis and graphical representation of the quantified luminescence intensity data.

## 3. Results

### 3.1. The Wild-Type and Optimized Fungal Luciferase Genes Were Separately Transformed into H. marmoreus

**Figure 1:**
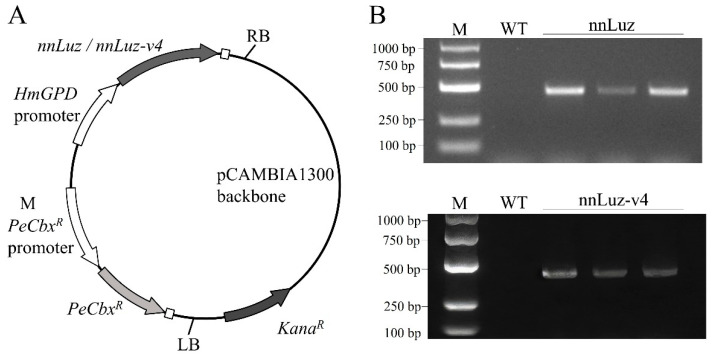
The binary vectors containing the wild-type fungal luciferase gene *nnLuz* or the optimized luciferase gene *nnLuz-v4* were separately transformed into *H. marmoreus*. (**A**) The T-DNA region (between LB and RB) contains the *PeCbx^R^* and *nnLuz/nnLuz-v4* expression cassettes. The *P. eryngii*-derived carboxin resistance gene (*PeCbx^R^*) was driven by its native promoter, and the *nnLuz* or *nnLuz-v4* gene was driven by the constitutive *H. marmoreus* GPD promoter. (**B**) PCR detection results of three independent *nnLuz* transformants and three independent *nnLuz-v4* transformants confirmed successful integration of the exogenous gene into the *H. marmoreus* genome. Genomic DNA from different transformed strains was used as template. Lane M: DNA molecular weight marker; lane WT: negative control PCR using wild-type *H. marmoreus* genomic DNA as template.

Transformation of *H. marmoreus* arthroconidia with both the *nnLuz* and *nnLuz-v4* binary vectors yielded carboxin-resistant colonies. PCR analysis of genomic DNA from representative transformants confirmed successful integration of the *nnLuz* or *nnLuz-v4* transgene in all carboxin-resistant colonies tested, indicating that both fungal luciferase genes were stably integrated into the *H. marmoreus* genome. The PCR detection results of three independent *nnLuz* transformants and three independent *nnLuz-v4* transformants are shown in Figure 1.

### 3.2. NnLuz Far Outperforms NnLuz-v4 in Luminescence Intensity

**Figure 2:**
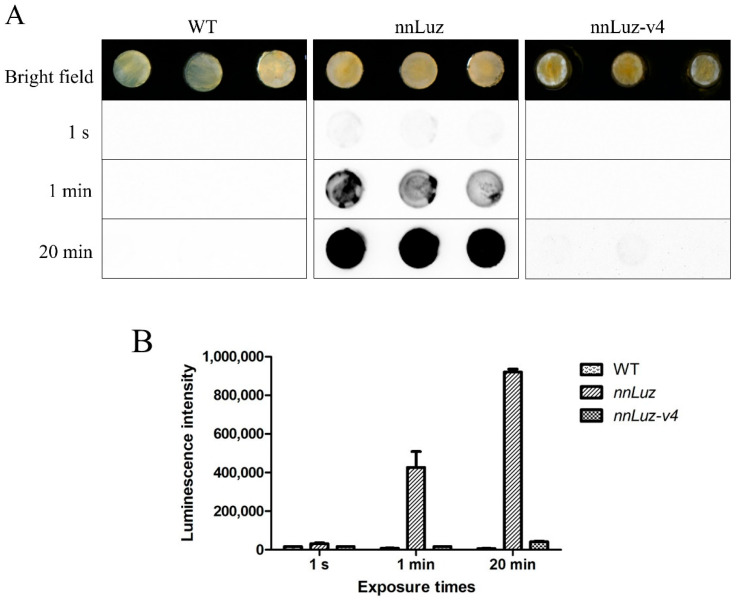
Fungal luciferase activity in transformed *H. marmoreus*. (**A**) Luminescence signals in mycelial plugs of wild-type and three independent transformants detected using the Tanon 5200 system with varying exposure times in the dark. First row: bright-field images of different strains. Rows 2–4: images after luciferin addition with exposure times of 1 s, 1 min, and 20 min, respectively. (**B**) Quantification of luminescence intensity using ImageJ and statistical analysis using GraphPad Prism 9. The wild-type background signal was very low; *nnLuz-v4* transformants showed detectable signal only after 20 min of exposure; *nnLuz* transformants exhibited significantly stronger luminescence than *nnLuz-v4* transformants.

The luminescence signals in mycelial plugs of wild-type and three independent transformants were detected using the Tanon 5200 automatic chemiluminescence image analysis system with varying exposure times in the dark. The luminescence signal of transformants containing the *nnLuz* gene could be detected with just a 1-s exposure. After a 1-min exposure, the luminescence signal on the entire mycelial surface could be clearly and stably detected. The signal became saturated after 20 min of exposure. In contrast, the wild-type *H. marmoreus* control strain still showed no luminescence signal even after 20 min of exposure. Interestingly, the previously published optimized luciferase gene version *nnLuz-v4*, despite significantly enhancing bioluminescence intensity in plants, yeast, and mammalian cells [7], performed much worse in *H. marmoreus* than the wild-type version *nnLuz*. Transformants carrying the *nnLuz-v4* gene showed no signal after a 1-min exposure, and only a weak signal could be detected after 20 min of exposure (Figure 2A).

We used ImageJ software to quantify the luminescence intensity of the wild-type *H. marmoreus*, three independent *nnLuz* transformants, and three independent *nnLuz-v4* transformants in images taken after the addition of luciferin. Then, we performed statistical analysis on the quantified data using GraphPad Prism 9 software. The results showed that the background signal of the wild-type strain was very low, that *nnLuz-v4* transformants became detectable only after 20 min of exposure, and that the luminescence signal of *nnLuz* transformants was significantly stronger than that of *nnLuz-v4* transformants. (Figure 2B).

## 4. Discussion

In this study, we report the first successful expression of a fungal luciferase gene—the *nnLuz* gene from the fungal bioluminescence pathway of *N. nambi*—in the edible mushroom *H. marmoreus*. Our results demonstrate that the expressed luciferase is enzymatically active and produces bioluminescence that is readily detectable using a CCD imaging system, with no background luminescence observed in control cultures. These findings establish the feasibility of deploying the fungal luciferase gene as a highly sensitive, autoluminescent reporter in cultivated edible fungi.

Unexpectedly, the wild-type *nnLuz* sequence consistently outperformed the optimized variant *nnLuz-v4* in terms of luminescence intensity and signal yield under identical experimental conditions. This result implies that codon optimization may not confer benefit—and may even prove detrimental—when the host and donor organisms are phylogenetically closely related basidiomycete fungi. The native codon usage pattern may preserve co-translational folding pathways that are essential for the proper enzymatic function of the fungal luciferase.

Previous studies achieved auto-luminescence in plants by introducing all four genes of the fungal FBP. However, the substrate of the FBP, caffeic acid, is a product of lignin metabolism in plants. Edible mushrooms do not necessarily produce abundant caffeic acid in their metabolic repertoire. Therefore, even if all four FBP genes were transformed into edible mushrooms, auto-luminescence might not be realized.

Firefly luciferase is widely used in genetic research and also requires the exogenous addition of luciferin. However, its application in fungi has rarely been reported. This may be due to poor performance arising from large species differences or potential toxicity after transformation. As a reporter derived from basidiomycetes, the fungal luciferase gene should be more suitable for use in edible fungi. In this study, we transformed *nnLuz* and *nnLuz-v4* into *H. marmoreus* and observed no growth differences between transformants and the wild-type strain.

The successful establishment of a luminescent reporter in *H. marmoreus* opens new avenues for genetic research in edible mushrooms. Unlike GFP-based reporters, this system requires no external excitation light source, generates no background luminescence, and can be coupled with simple, cost-efficient CCD detection. Potential applications include promoter characterization, real-time monitoring of gene expression during different developmental stages (mycelial growth, primordium formation, and fruiting body maturation), and the construction of biosensors responsive to environmental or nutritional cues. This work represents a foundational step towards the wider adoption of fungal luciferase gene-based genetic tools in mushroom science and may accelerate breeding programmes aimed at improving agronomically important traits such as yield, stress tolerance, and nutritional quality.

## Data Availability

The original contributions presented in this study are included in the article. Further inquiries can be directed to the corresponding authors.
